# New process for production of fermented black table olives using selected autochthonous microbial resources

**DOI:** 10.3389/fmicb.2015.01007

**Published:** 2015-09-24

**Authors:** Maria Tufariello, Miriana Durante, Francesca A. Ramires, Francesco Grieco, Luca Tommasi, Ezio Perbellini, Vittorio Falco, Maria Tasioula-Margari, Antonio F. Logrieco, Giovanni Mita, Gianluca Bleve

**Affiliations:** ^1^Consiglio Nazionale delle Ricerche-Istituto di Scienze delle Produzioni Alimentari, Unità Operativa di LecceLecce, Italy; ^2^Associazione “Olivicoltori di Puglia,”Lecce, Italy; ^3^Agricola Nuova Generazione Soc. Coop.Martano, Lecce, Italy; ^4^Section of Food Chemistry, Department of Chemistry, University of IoanninaIoannina, Greece; ^5^Consiglio Nazionale delle Ricerche-Istituto di Scienze delle Produzioni AlimentariBari, Italy

**Keywords:** table olives, yeast, lactic acid bacteria, starter, fermentation

## Abstract

Table olives represent one important fermented product in Europe and, in the world, their demand is constantly increasing. At the present time, no systems are available to control black table olives spontaneous fermentation by the Greek method. During this study, a new protocol for the production of black table olives belonging to two Italian (Cellina di Nardò and Leccino) and two Greek (Kalamàta and Conservolea) cultivars has been developed: for each table olive cultivar, starter-driven fermentations were performed inoculating, firstly, one selected autochthonous yeast starter and, subsequently, one selected autochthonous LAB starter. All starters formulation were able to dominate fermentation process. The olive fermentation was monitored using specific chemical descriptors able to identify a first stage (30 days) mainly characterized by aldehydes; a second period (60 days) mainly characterized by higher alcohols, styrene and terpenes; a third fermentation stage represented by acetate esters, esters and acids. A significant decrease of fermentation time (from 8 to 12 months to a maximum of 3 months) and an significant improvement in organoleptic characteristics of the final product were obtained. This study, for the first time, describes the employment of selected autochthonous microbial resources optimized to mimic the microbial evolution already recorded during spontaneous fermentations.

## Introduction

Table olives represent one important fermented product in Europe and their demand is nowadays increasing throughout both EU and the world and producer countries are also the most important consumers (International Olive Council (IOC), 2014). Kalamàta and Conservolea, together with Manzanilla, Sevillana and Hojiblanca, Bella di Cerignola and Ascolana Tenera, are the most important table olive varieties (Anonymous, 2003).

The most technological procedures employed in the industrial production of table olives are: (i) the Spanish-system (green olives), (ii) the Californian-system (black oxidized olives) and (iii) the Greek-system (black olives in brine) (Garrido-Fernández, 1997). In particular, the last one is used for the production of Conservolea, Kalamàta, Leccino and Cellina di Nardò table olives cultivars. Conservolea and Kalamàta represent the most economically important cultivars in Greece for domestic and foreign market (Garrido-Fernandez et al., 1997); whereas, among Italian table olive cultivars, Leccino is the most important olive variety used for dual-purpose in the world (Vossen, 2007) and Cellina di Nardò is a very promising table olive cultivar in Salento (Apulia, Southern Italy), highly appreciated for its peculiar organoleptic and sensorial features (Bleve et al., 2015a).

Bleve et al. (2014, 2015a) studied the evolution of several compounds (sugars, organic acids, alcohols, mono and polyphenols and volatiles) associated with yeasts and bacteria fermentative metabolism of during the spontaneous fermentation process of these Italian and Greek olive cultivars. By this approach, chemical compounds deriving by microbiological activities during the fermentation process were proposed as chemical descriptors to monitor the fermentation process.

Currently, black olives and several cultivars of green olives are industrially produced by spontaneous fermentation which are difficult to be monitored and controlled (De Castro et al., 2002; Tassou et al., 2002; Alvarez et al., 2003; Abriouel et al., 2011).

In order to control the fermentation process and to improve the quality of the final product, the use of starter cultures of *Lactobacillus plantarum* and *L. pentosus* has already been tested (De Castro et al., 2002; Leal-Sánchez et al., 2003; Panagou et al., 2003, 2008; Marsilio et al., 2005; Servili et al., 2006; Sabatini and Marsilio, 2008). Also yeasts are known to be able to improve the organoleptic properties by the production of desirable metabolites and volatile compounds (Garrido Fernández et al., 1995; Arroyo-López et al., 2008). Furthermore, yeasts are able to enhance the growth of Lactic Acid Bacteria (LAB) (Tsapatsaris and Kotzekidou, 2004; Segovia Bravo et al., 2007) and to biodegrade phenolic compounds (Ettayebi et al., 2003). Indeed, a possible role of yeasts as starters have been recently proposed for production of table olive (Arroyo-López et al., 2008, 2012a,b; Bevilacqua et al., 2013; Bonatsou et al., 2015). We previously reported that in several cultivars of black table olives yeasts were present during the fermentation, whereas LAB resulted associated only to the last stage of the process produced (Bleve et al., 2014, 2015a). Therefore, a specific protocol was set up to select LAB and yeast strains from Conservolea, Kalamàta, Leccino and Cellina di Nardò to be used as autochthonous starters for table olives production.

On the basis of the results previously obtained, the main scopes of the present work were (i) to study the microbiological, biochemical and chemical profiles associated with pilot-scale fermentations driven by a sequential inoculum of selected autochthonous yeast and LAB starter in comparison with natural fermentations, (ii) to validate the use of previously proposed (Bleve et al., 2014, 2015a) fermentation by-products as a functional tool for process monitoring. For the first time in our knowledge a mixed yeast/LAB starter, specific for each analyzed table olive cultivar, was used for olive production, thus allowing to obtain in a short time period (90 days) a fermented final product with improved organoleptic characteristics.

## Materials and methods

### Microbial strains and growth conditions

The yeast strains used in this study were: *Saccharomyces cerevisiae* LI 180-7 isolated from Leccino, *Pichia anomala* CL 30-29 isolated from Cellina di Nardò, *S. cerevisiae* KI 30-16 isolated from Kalamàta, *Debaryomyces hansenii* A 15-44 isolated from Conservolea. The LAB employed in this study were: *L. plantarum* L 180-11 isolated from Leccino, *L. plantarum* C 180-34 isolated from Cellina di Nardò, *Leuconostoc mesenteroides* K T5-1 isolated from Kalamàta, *L. plantarum* A 135-5 isolated from Conservolea. Yeasts were grown in YPD medium (yeast extract 1% w/v, meat peptone 2% w/v, glucose 2% w/v and agar 2% w/v) and incubated at 28°C for 24–48 h. LAB were grown in Man, Rogosa and Sharpe Agar (MRS, LABM, UK) at 28°C under anaerobic conditions for 48–72 h.

Yeast isolates used as starter candidates were initially inoculated on YPD broth and then they were adapted to pH and salt conditions. Cultures were adapted to specific salt and pH conditions: they were firstly grown on YPD broth containing 2% NaCl (wt/vol) and at pH 4.5 and then they were inoculated on YPD broth containing 4% NaCl (wt/vol) and at pH 4.5. Finally, (about 3–5 L) of a culture suitable to inoculate about 200 kg of olives were produced on YPD broth containing 5% NaCl (wt/vol) and at pH 4.5. LAB isolates used as starters were initially inoculated on MRS broth and then they were adapted to pH and salt conditions. Cultures were firstly grown on MRS broth containing 2% NaCl (wt/vol) and at pH 4.5 and then they were inoculated on MRS broth containing 4% NaCl (wt/vol) and at pH 4.5. Finally, (about 5 L) of a culture suitable to inoculate about 200 kg of olives was produced on MRS broth containing 5% NaCl (wt/vol) and at pH 4.5.

### Pilot-scale olives fermentations

The pilot-scale fermentations were performed in triplicate on olive samples of Cellina di Nardò, Leccino, Kalamàta and Conservolea cultivars at an industrial plant located in Salento (Southern Italy). Samples of healthy black olives (150 kg) were washed, selected for a 10–12 mm caliber and placed in plastic vessels of 200 kg capacity filled with 50 L of 12% NaCl (wt/vol) for Cellina di Nardò and Leccino and of 8% NaCl (wt/vol) for Kalamàta and Conservolea cultivars. The olives were allowed to ferment at ambient temperature, in presence or absence of starter additions.

Starter cultures, consisting of previously selected yeast and LAB strains (Bleve et al., 2014, 2015a) were used to drive olive pilot-scale fermentations, by a sequential inoculation strategy, according to the following scheme: Leccino olives inoculated firstly with *S. cerevisiae* LI 180-7 and then with *L. plantarum* L 180-11; Cellina di Nardò olives inoculated firstly with *Pichia anomala* CL 30-29 and then with *L. plantarum* isolate C 180-34; Kalamàta olives firstly inoculated with *S. cerevisiae* KI 30-16 and then with *L. mesenteroides* K T5-1; Conservolea olives firstly inoculated with *D. hansenii* A15-44 and then with *L. plantarum* A135-5.

The strains *S. cerevisiae* LI 180-7, *S. cerevisiae* isolate KI 30-16, *L. plantarum* isolate L 180-11, and *L. mesenteroides* K T5-1 were deposited into the DSMZ collection and the codes DSMZ27800, DSMZ27801, DSMZ27925, and DSMZ27926 were respectively assigned (Bleve et al., 2013, 2015a).

Pure cultures of candidate yeast and LAB starters, each corresponding to about 10^6^ CFU/ml, were adapted to the high salinity environment [5% NaCl (wt/vol)] and low pH (pH 4.5) and then used to inoculate 200 kg of washed olives. For each inoculated fermentation, a spontaneous one was carried out as control. Thus, starter-driven started by inoculating the selected olive-cultivar-specific yeast starter to each table olive cultivar. Then, the time of addition of cultivar-specific LAB (63th day after yeast inoculation) was determined by the appearance, in the brine and in the olives, of definite compounds (i.e., higher alcohols, styrene, terpenes) indicating the end of yeast fermentation activities (Bleve et al., 2014, 2015a). Olive fermentation progress was considered ended when other specific chemical descriptors, consisting of esters and acetate esters, were detectable (Bleve et al., 2014, 2015a).

### Microbiological analyses

During fermentation, at different time points (0, 7, 14, 21, 28, 35, 42, 49, 56, 63, 70, 77, 84, 90 days) the following parameters were evaluated: pH, salinity, temperature and mold layer formation. Olives and brines were collected at each fermentation time point and, after, incubation for 30 min at 200 rpm, aliquots of brines, diluted with sterile 100% glycerol, were stored at −80°C for further analysis. LAB, *Enterobacteriaceae* and yeasts associated to olives and brines were quantified serially diluting samples with 0.1% (wt/vol) peptone water and applying them onto MRS, VRBGA and Sabouraud media agar plates respectively added with nystatin, ampicillin and kanamycin. MRS plates were incubated at 28°C under anaerobic conditions for 48–72 h; VRBGA plates were incubated at 37°C for 18–24 h; Sabouraud Dextrose Agar plates were incubated at 25°C for 2–4 days.

Dominance test was performed on yeasts isolated after 28 and 56 days of fermentation and on LAB isolated after the inoculum (63th day) and at the end of the fermentation process (90th day). Yeasts and LAB were isolated by plating 10 fold dilutions of brine sample in order to obtain 100–200 colonies per 90 mm diameter plate. For each sampling times indicated above, 40 colonies were randomly selected from the same dilution from the agar plates (specific for LAB and yeasts) and processed for (GTG)(5)-rep-PCR analyses.

### Molecular analyses

The dominance of inoculated yeast and LAB strains was tested by the (GTG)(5)-rep-PCR fingerprinting technique. Total genomic DNA from the yeast strains was prepared according to the method used by De Benedictis et al. (2011), whereas total LAB DNA was extracted as described by Wilson (2001). For both yeast and LAB, amplification reactions were performed in a final volume of 50 μl containing 100 ng of genomic DNA, 10 mM Tris HCl, 50 mM KCl, 1.5 mM MgCl2, 2 mM of each dNTP, 0.8 μM of primer (GTG)_5_ (Cadez et al., 2002) and 1 U of Taq DNA polymerase (Euroclone, Italy). The thermal cycler was programmed for 40 cycles of 30 s at 94°C, 30 s primer annealing at 42°C, 2 min at 72°C with a final extension cycle of 10 min at 72°C. The amplified DNA products were visualized by agarose gel electrophoresis (Bleve et al., 2006).

### Soluble sugar, organic acid, and phenols extraction from the olive fruits

Twenty olive fruits were pitted, finely chopped and dehydrated to constant weight by Christ ALPHA 2–4 LSC freeze-dryer (Martin Christ Gefriertrocknungsanlagen GmbH, Osterode am Harz, Germany). Dried olive fruits were ground in a laboratory ultra centrifugal mill (ZM200, Retsch GmbH, Haan, Germany).

Soluble sugars were extracted from 0.05 g powder of olives, as described by Eris et al. (2007) with slight modifications, mixed with 2 ml of 80% (v/v) of ethanol, stirred for 1 min and incubated at 85°C for 1 h in a water bath. The supernatant was recovered by centrifugation at 5000 × g for 10 min. This procedure was repeated four times for 1 h, 30 min, 15 min (twice). The ethanolic solutions were collected and the combined extracts were evaporated to dryness at 55°C.

Organic acids were extracted from 0.05 g powder olives, as described by Günç Ergönül and Nergiz (2010), mixed with 10 mL water-methanol solution (75:25 v/v) and stirred for 10 min. The supernatant was recovered by centrifugation at 5000 × g for 10 min. This procedure was repeated three times and the supernatants were collected and pooled.

Phenolic compounds were extracted, from 1 g powder of olives, as described by Hajimahmoodi et al. (2008), mixed with 5 ml of 50% (v/v) of methanol and stirred for 15 min. Supernatant was recovered by centrifugation at 12,000 rpm for 15 min. This procedure was repeated six times. The organic solutions were collected and the combined extracts were evaporated to dryness. The residue was dissolved in a final volume 3 mL of methanol.

### HPLC analyses

HPLC analysis was carried out on an Agilent 1100 series HPLC (Agilent Technologies, Waldbronn, Germany) equipped with a photodiode array detector (for phenols and organic acids analysis) and RID-10 A refractive index detector (for sugars and alcohols analysis). Phenols compounds were achieved according to Li et al. (2008), slightly modified by using a Phenomenex Luna 5 μm C18 (2) 100 Å column (250 × 4.6 mm). The mobile phase consisted of the acetonitrile (solution A) and 1% (v/v) phosphoric acid in water (solution B) using a gradient elution of 5–15% B, 0–30 min; 15–23% B, 30–45 min; 23–50% B, 45–55 min; 50–70% B, 55–65 min; and then returned to initial condition for a 10 min re-equilibration. The analysis was carried out at a flow rate of 1 mL/min with the detection wavelength set at 280, 295 and 320 nm. All phenol compounds were identified by comparing their retention times and UV-vis spectra to authentic standards. Phenols in the brine were directly analyzed in the HPLC system after a filtration through 0.45 μm filter before injection.

Separation of sugars, organic acids and alcohols were simultaneously carried out, according to De Benedictis et al. (2011), on an Aminex HPX-87H column (300 × 7.8 mm) (Bio-Rad) and kept at 55°C. Photodiode array detector was set at 210 nm. As eluent was used 0.045 N phosphoric acid with 6% acetonitrile (v/v) at flow rate 0.3 mL/min. Sugars, organic acids and alcohols in the brine were directly analyzed in the HPLC system after a filtration through 0.45 μm filter before injection.

### GC/MS analysis

The extraction of volatile molecules was carried out by SPME (solid phase micro extraction) procedure, and analyzed by using gas chromatography/mass spectrometry (GC-MS) according to Bleve et al. (2015a) and Malheiro et al. (2011). The GC–MS system used was an Agilent 6890N GC coupled to an Agilent 5973 mass spectrometer, the molecules separation was performed by a DB-WAX column (60 m^*^0.25 mm i.d.^*^0.25 mm film thickness). The MS detector operated in scan mode (mass range 30–350). Semi-quantitative analysis was carried out by internal standard method.

### Statistical analysis

Chemical data are presented as mean values ± standard deviation of three independent experiments. One-Way factor analysis of variance (ANOVA) followed by Duncan's *post-hoc* comparisons tests was performed to establish significant differences for volatile compounds and Tukey's test for sugars, organic acids and alcohols. The level of significance was set at *P* < 0.05. Principal component analysis (PCA) was used to compare volatile classes of compounds during fermentations. All statistical analyses were carried out using the STATISTICA 7.0 software (StatSoft software package, Tulsa, OK, USA).

## Results

### Pilot-scale olives fermentations

Autochthonous yeast and LAB isolates used in this study were previously obtained by the following procedure: (i) isolation from spontaneous fermentations of Leccino, Cellina di Nardò, Kalàmata and Conservolea table olives; (ii) step-by-step selection on model brines, presence of beta-glucosidase activity, absence of amino acids decarboxylation activities, presence of protease and lipase activities. At the end of this procedure, yeast and LAB isolates carrying the most promising technological and safety characteristics were chosen to perform pilot-scale fermentation experiments. Leccino olives were inoculated with *S. cerevisiae* LI 180-7/ *L. plantarum* L 180-11; Cellina di Nardò olives inoculated with *Pichia anomala* CL 30-29/ *L. plantarum* isolate C 180-34; Kalamàta olives inoculated *S. cerevisiae* KI 30-16/ *L. mesenteroides* K T5-1; Conservolea olives inoculated with *D. hansenii* A15-44/ *L. plantarum* A135-5.

**Figure 1:**
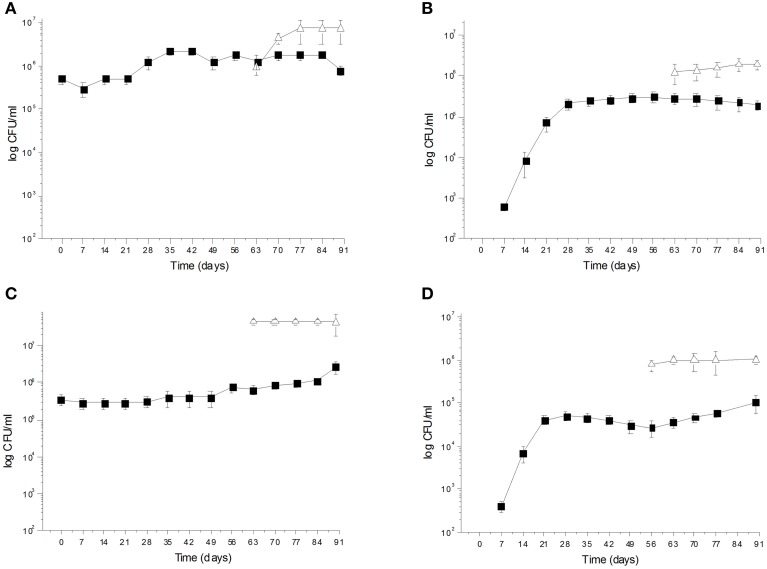
**(A)** *Saccharomyces cerevisiae* LI 180-7 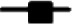 and *Lactobacillus plantarum* LI 180-11 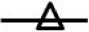 total counts (Log CFU/ml) in Leccino fermented table olives; **(B)** Yeast 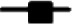 and LAB 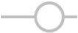 total counts (Log CFU/ml) of Leccino spontaneously fermented table olives; **(C)** *Pichia anomala* CL 30-29 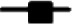 and *L. plantarum* C 180-34 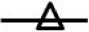 total counts (Log CFU/ml) in Cellina di Nardò fermented table olives; **(D)** Yeast 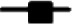 and LAB 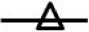 total counts (Log CFU/ml) of Cellina di Nardò naturally fermented table olives.

**Figure 2:**
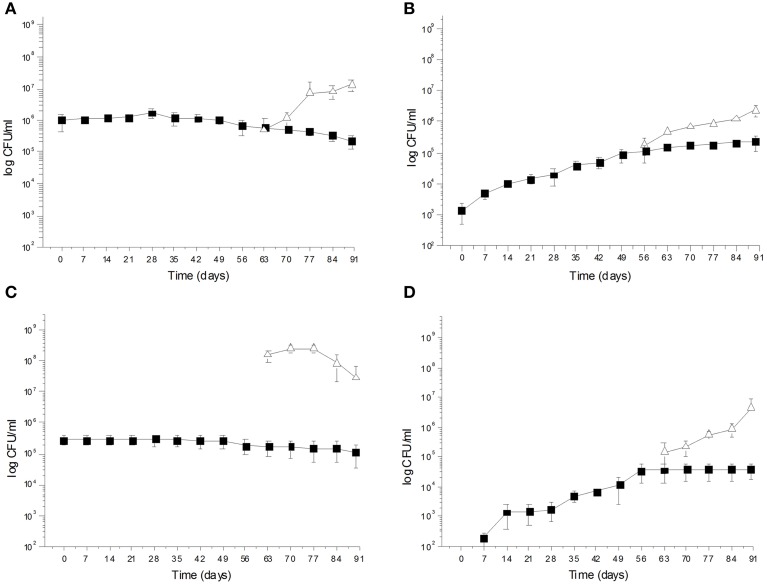
**(A)** *Saccharomyces cerevisiae* KI 30-16 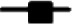 and *Leuconostoc mesenteroides* K T5-1 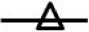 total counts (Log CFU/ml) in Kalamàta fermented table olives; **(B)** Yeast 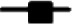 and LAB 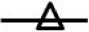 total counts (Log CFU/ml) of Kalamàta spontaneously fermented table olives; **(C)** *Debaryomyces hansenii* A 15-44 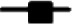 and *L. plantarum* A 135-5 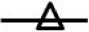 total counts (Log CFU/ml) in Conservolea fermented table olives; **(D)** Yeast 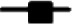 and LAB 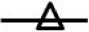 total counts (Log CFU/ml) of Conservolea spontaneously fermented table olives.

During spontaneous olive fermentations, yeasts were present during the fermentation process, whereas LAB started to be detectable in the last 30 days performing lactic fermentation (Bleve et al., 2014, 2015a; Figures 1B,D, 2B,D). In starter-driven fermentations, yeast inocula maintained or increased their initial count (2 × 10^5^–10^6^ CFU/ml) and they were present throughout the process (Figures 1A,C, 2A,C). The dominance of inoculated strains was confirmed by the (GTG)(5)-rep-PCR fingerprinting technique. Table 1 shows the percentage of survival of yeats and LAB along the fermentations. In particular, at day 56 (corresponding to the end of yeast fermentation) strain *S. cerevisiae* LI 180-7 for Leccino was able to dominate with a predominance of 60% in total yeast population, the strain *Pichia anomala* isolate CL 30-29 for Cellina di Nardò dominated with a predominance of 100%, the strain *S. cerevisiae* isolate KI 30-16 for Kalamàta dominated with a predominance of 100% and finally the strain *D. hansenii* A15-44 for Conservolea dominated with a predominance of 70% (Table 1).

**Table 1:** **Percentage survival of inoculated yeast and LAB strains during the process of fermentation**.

**Day**	**Leccino**		**Cellina di Nardò**		**Kalamàta**		**Conservolea**	
	***S. cerevisiae* LI-180-7 (%)**	***L. plantarum* L180-11 (%)**	***P. anomala* CL 30-29 (%)**	***L. plantarum* C180-34 (%)**	***S. cerevisiae* KI 30-16 (%)**	***L. mesenteroides* K T5-1 (%)**	***D. hansenii* A 15-44 (%)**	***L. plantarum* A 135-5 (%)**
0	70	–	70	–	70	–	70	–
28	90	–	85	–	90	–	80	–
56	60	–	100	–	100	–	70	–
63	60	65	100	60	100	70	70	80
90	50	80	60	60	60	80	40	80

After inoculation, LAB concentration maintained or increased to 10^7^- 5 × 10^7^ CFU/ml at the end of the process (Figures 1A,C, 2A,C). All autochthonous LAB strains were able to dominate the processes (Table 1): at the day 90 the strain *L. plantarum* isolate L 180-11 for Leccino, *L. plantarum* isolate C 180-34 for Cellina di Nardò, *L. mesenteroides* isolate K T5-1 for Kalamàta, *L. plantarum* A135-5 for Conservolea dominated with a predominance of 80, 60, 80 and 80%, respectively (Table 1). *Enterobacteriaceae* resulted undetectable in brines derived from all olives cultivars during natural and starter-driven fermentation processes (data not shown).

### Physic-chemical dynamics of brines

Leccino and Kalamata fermentations on one side and Cellina di Nardò and Conservolea fermentations from another, showed in pairs the same pattern of temperature variation, because their different processing time (Leccino and Kalamàta late November mid-February and Cellina di Nardò and Conservolea mid-December mid-March) (Supplementary Figures 1, 2). In all fermentations, the pH values quickly decreased (3.5–4.5) within the first 10–15 days of fermentation and, after the 90th, they maintained at their minimum values (Supplementary Figures 1, 2). Salinity was checked throughout the fermentations to maintain it almost stable about a value of 10% (w/v) for Leccino and Cellina di Nardò and of 8% (w/v) for Kalamàta and Conservolea (Supplementary Figures 1, 2).

Glucose was completely consumed in Cellina di Nardò, Kalamàta and Conservolea fermentations, whereas a limited amount remained in Leccino olives (7.2 mg/g DW) (Table 2). Except in Kalamàta olives, where glucose was totally metabolized, in control fermentations of all the other three cultivars, glucose concentrations was reduced to 15–9.2 mg/g DW. The fructose and sucrose contents showed a decrease during both the control and starter-driven fermentations of table olive cultivars (Table 2). In all the starter-driven fermentation samples, ethanol concentration in brines increased gradually with time and reached a final concentration of about 0.61–2.28 g/L. With the exception of Cellina di Nardò, where glycerol was undetectable in brines, glycerol content increased during fermentations to 0.11–1.38 g/L (Table 2). The level of total organic acids in starter-driven fermentation brines was found to be higher or comparable to the corresponding natural control fermentations and these compounds, particularly lactic and acetic acids, increased during fermentation (Table 2). Oleuropein content in drupes was undetectable in drupes and brines in all fermentations, except for Cellina di Nardò drupes where this phenol compound ranged from 20 to 3 mg/g DW after treatment with starter. In all starter-treated samples, the main phenolic compounds detected in brines were hydroxytyrosol (ranging about 398 and 1050 mg/L) and tyrosol (ranging from 68 to 206 mg/L). Cellina di Nardò and Leccino brines, naturally or starter inoculated, were rich in verbascoside and caffeic acid (data not shown).

**Table 2:** **Sugar, organic acid and alcohol evolution in Leccino, Cellina di Nardò, Kalamàta and Conservolea drupes (A) and brines (B) during spontaneous and starter-driven fermentation process**.

	**Leccino**			**Cellina di Nardò**			**Kalamàta**			**Conservolea**		
		**CTRL**	**Starter**		**CTRL**	**Starter**		**CTRL**	**Starter**		**CTRL**	**Starter**
	**mg/g DW**											
	**0 days**	**90 days**		**0 days**	**90 days**		**0 days**	**90 days**		**0 days**	**90 days**	
**A**												
**ORGANIC ACID**												
Citric acid	20.8 ± 2.5	5.7 ± 0.1^a^	4.3 ± 0.1^b^	5.8 ± 0.3	6.3 ± 1.3^a^	7.6 ± 0.6^a^	24.1 ± 3.2	ND	ND	39.5 ± 1.5	5.8 ± 0.3^a^	5.8 ± 0.3^a^
Tartaric acid	19.1 ± 1.5	9.5 ± 0.7^a^	7.8 ± 0.1^b^	15.6 ± 0.8	8.8 ± 0.8^a^	9.3 ± 1.3^a^	26.8 ± 3.9	ND	ND	26.5 ± 0.9	4.1 ± 0.4^a^	2.5 ± 0.1^b^
Malic acid	12.1 ± 0.9	5.8 ± 0.3^a^	4.1 ± 0.1^b^	7.8 ± 1.1	7.1 ± 0.1^a^	6.5 ± 1.1^a^	13.0 ± 1.4	1.2 ± 0.2^a^	0.1 ± 0.01^b^	12.6 ± 0.8	2.9 ± 0.7^a^	2.0 ± 0.1^a^
Succinic acid	7.7 ± 0.1	7.4 ± 0.8^a^	4.5 ± 0.1^b^	ND	14.7 ± 2.4^a^	7.9 ± 0.1^b^	7.1 ± 0.9	ND	ND	3.4 ± 0.6	ND	ND
Lactic acid	19.1 ± 1.3	6.4 ± 0.6^a^	12.2 ± 2.8^b^	28.8 ± 1.1	37.6 ± 0.6^a^	16.9 ± 1.3^b^	24.2 ± 3.6	16.5 ± 1.8^a^	16.0 ± 2.8^a^	15.2 ± 0.3	15.2 ± 0.3^a^	17.9 ± 0.7^b^
Acetic acid	39.1 ± 1.4	28.8 ± 1.1^a^	20.5 ± 0.3^b^	4.7 ± 0.4	15.8 ± 2.8^a^	27.4 ± 0.6^b^	144.5 ± 12.0	1.5 ± 0.1^a^	15.8 ± 0.3^b^	19.2 ± 1.1	ND	ND
**SUGAR**												
Glucose	32.1 ± 2.9	10.7 ± 0.2^a^	7.2 ± 0.1^b^	32.7 ± 0.7	15.0 ± 1.1^a^	0.10 ± 0.01^b^	33.5 ± 2.1	ND	ND	3.0 ± 0.3	9.2 ± 0.6^a^	0.1 ± 0^b^
Fructose	32.0 ± 2.8	2.8 ± 1.1^a^	1.6 ± 0.1^a^	ND	ND	ND	26.5 ± 0.7	ND	ND	46.9 ± 4.9	ND	ND
Sucrose	7.0 ± 1.4	3.2 ± 0.3^a^	1.9 ± 0.1^b^	ND	ND	ND	5.5 ± 0.7	ND	ND	18.7 ± 1.2	ND	ND
**ALCOHOL**												
Glycerol	ND	ND	ND	ND	ND	ND	ND	ND	ND	ND	ND	ND
Ethanol	ND	ND	ND	ND	ND	ND	ND	ND	ND	ND	ND	ND
**B**												
**ORGANIC ACID**												
Citric acid	ND	1.68 ± 0.02^a^	0.90 ± 0.01^b^	ND	3.10 ± 0.02^a^	2.97 ± 0.09^a^	ND	0.01 ± 0.0^a^	0.19 ± 0.06^b^	ND	1.91 ± 0.09^a^	1.90 ± 0.01^a^
Tartaric acid	ND	1.46 ± 0.06^a^	1.06 ± 0.09^b^	ND	2.90 ± 0.12^a^	1.50 ± 0.04^b^	ND	1.06 ± 0.01^a^	0.92 ± 0.01^b^	ND	0.65 ± 0.04^a^	0.46 ± 0.03^a^
Malic acid	ND	0.60 ± 0.02^a^	0.72 ± 0.05^a^	ND	1.21 ± 0.01^a^	1.21 ± 0.06^a^	ND	0.28 ± 0.02^a^	0.57 ± 0.06^b^	ND	2.26 ± 0.18^a^	1.69 ± 0.01^a^
Succinic acid	ND	0.58 ± 0.03^a^	3.09 ± 0.07^b^	ND	0.53 ± 0.04^a^	1.71 ± 0.04^b^	ND	3.99 ± 0.03^a^	0.50 ± 0.04^b^	ND	0.76 ± 0.05^a^	0.76 ± 0.05^a^
Lactic acid	ND	0.74 ± 0.04^a^	2.79 ± 0.04^b^	ND	1.29 ± 0.01^a^	3.90 ± 0.01^b^	ND	3.84 ± 0.04^a^	2.49 ± 0.10^b^	ND	8.22 ± 0.70^a^	5.72 ± 0.04^b^
Acetic acid	ND	1.48 ± 0.08^a^	1.68 ± 0.07^b^	ND	0.10 ± 0.01^a^	0.11 ± 0.01^b^	ND	0.06 ± 0.0^a^	3.17 ± 0.19^b^	ND	6.41 ± 0.30^a^	5.01 ± 0.06^b^
**SUGAR**												
Glucose	ND	0.69 ± 0.04^a^	0.01 ± 0.0^b^	ND	1.75 ± 0.09^a^	0.30 ± 0.09^b^	ND	0.04 ± 0.0^a^	0.42 ± 0.04^b^	ND	ND	ND
Fructose	ND	0.50 ± 0.03^a^	0.46 ± 0.03^a^	ND	3.24 ± 0.02^a^	0.13 ± 0.01^b^	ND	0.05 ± 0.0^a^	0.29 ± 0.03^b^	ND	ND	ND
**ALCOHO**L												
Glycerol	ND	0.53 ± 0.04^a^	0.86 ± 0.05^b^	ND	ND	ND	ND	0.11 ± 0.01^a^	2.27 ± 0.09^b^	ND	1.38 ± 0.01^a^	0.14 ± 0.01^b^
Ethanol	ND	2.99 ± 0.99^a^	2.28 ± 0.08^b^	ND	2.23 ± 0.12^a^	1.50 ± 0.09^b^	ND	0.94 ± 0.09^a^	0.61 ± 0.05^b^	ND	1.60 ± 0.01^a^	2.50 ± 0.24^b^

*For each olive cultivar, used starter combinations are as reported in Table 1. Data are the mean ± standard deviation of three independent replicates (n = 3). Data were submitted to One-Way analysis of variance (ANOVA), Tukey's post hoc method was applied to establish significant differences between fermentations (p < 0.05). ND, not detected*.

a, b *The different letters indicate significant differences between fermentations (p < 0.05)*.

### Analysis of volatile compounds in olives and brines

**Figure 3:**
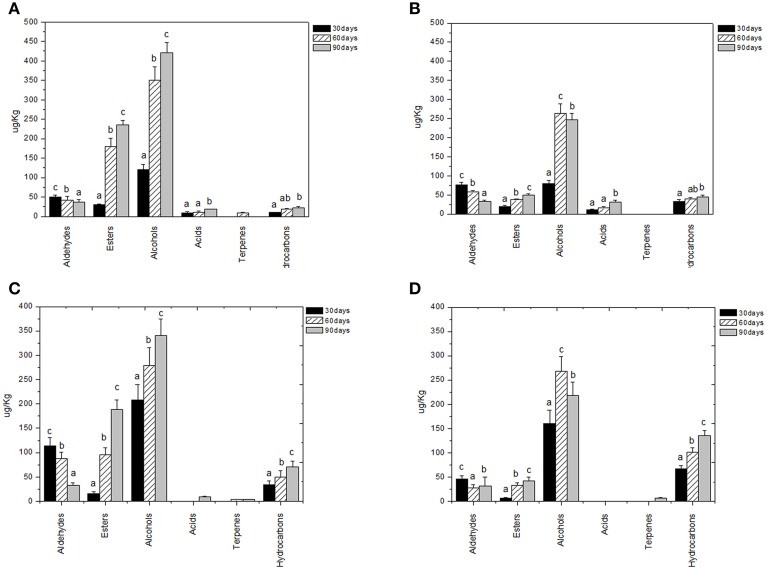
**Evolution of volatile compound classes of: Leccino drupes (A) during fermentation driven by starters yeast) and LAB and (B) during spontaneous fermentation process; Cellina di Nardò drupes (C) during fermentation driven by starters yeast and LAB and (D) during spontaneous fermentation process**. a,b,c: the different letters indicate significant differences among stage of fermentation in the same volatile classes (*p* < 0.05).

**Figure 4:**
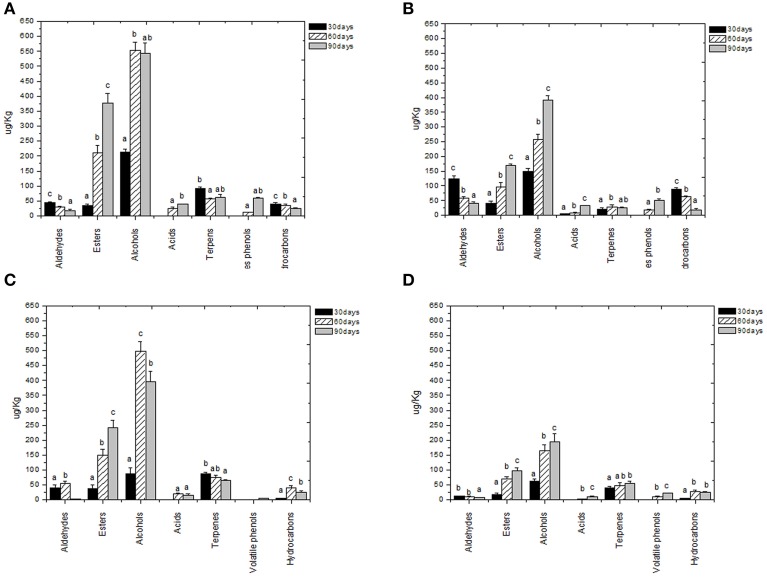
**Evolution of volatile compound classes of: Kalamàta drupes (A) during fermentation driven by starters yeast) and LAB and (B) during spontaneous fermentation process; Conservolea drupes (C) during fermentation driven by starters yeast and LAB and (D) during spontaneous fermentation process**. a,b,c: the different letters indicate significant differences among stage of fermentation in the same volatile classes (*p* < 0.05).

Volatile compounds detected and identified belonged to esters, aldehydes/ketones, terpenes alcohols, volatile phenols, lactones, acids, and hydrocarbons classes. The four table olive cultivars differed significatively in aldehydes content, and it showed a decrease in concentration during starter-driven fermentations in a manner comparable to what observed in the corresponding natural fermentations (Figures 3, 4; Supplementary Tables 1–4). Alcohol and ester contents increased during starter-driven fermentations, with higher concentrations in Leccino and Kalamàta than in Cellina di Nardò and Conservolea, and all higher than those produced in the corresponding spontaneous fermentations (Figures 3, 4; Supplementary Tables 1–4). No variation of terpenes and hydrocarbons content was detected between starter-driven and spontaneous fermentations. However, significant organic acid and volatile phenols concentrations were respectively found in starter-inoculated Cellina di Nardò and Conservolea fermentations (Figures 3, 4; Supplementary Tables 1–4).

### Principal component analysis (PCA)

**Figure 5:**
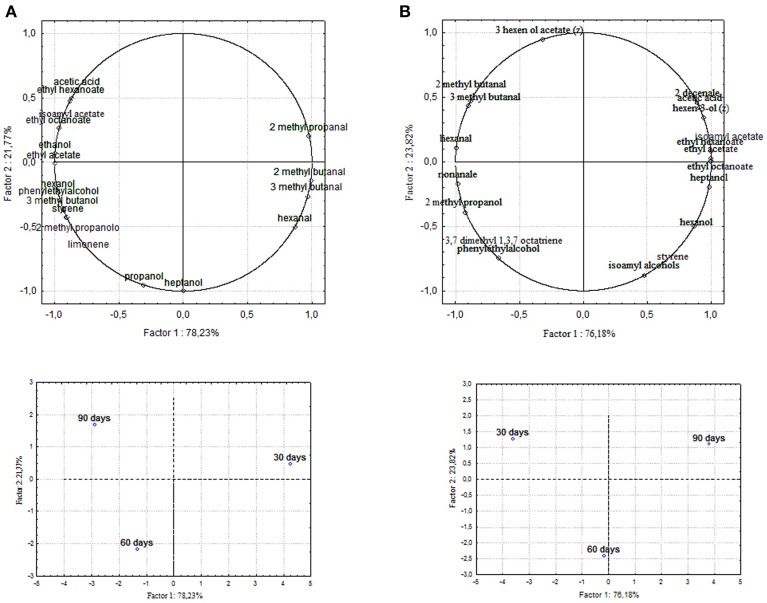
**PCA of volatile compounds associated with (A) Leccino and (B) Cellina di Nardò starter-driven fermented table olives**. PCA variables were the data obtained from the analysis of concentration and presence of volatile compounds at three different fermentation times. The figure displays the sample scores and variable loadings in the planes formed by PC1–PC2.

**Figure 6:**
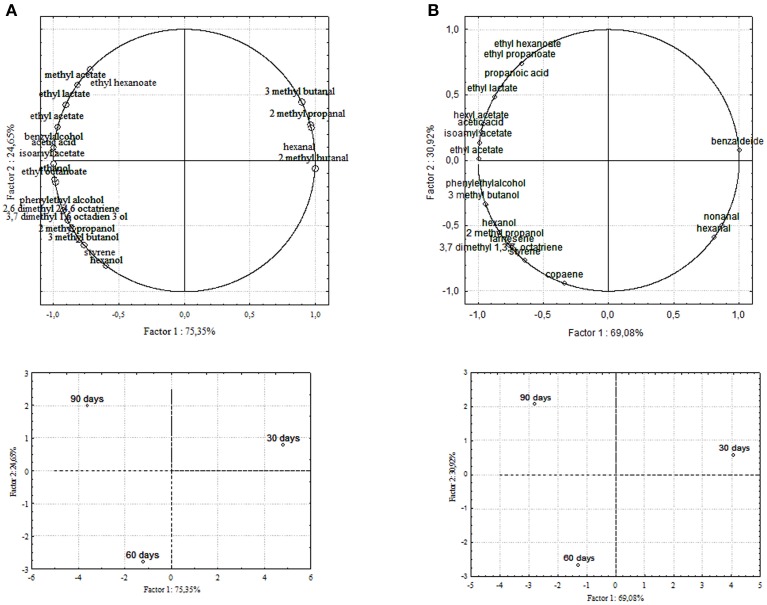
**PCA of volatile compounds associated with (A) Kalamàta and (B) Conservolea starter-driven fermented table olives**. Score plot of variables (concentration of volatile molecules) and three different fermentation times in the plane formed by the first two principal components (PC1 against PC2).

Chemical data were correlated with the activities of mixed starter microorganisms by a PCA analysis was carried out on the global SPME/GC-MS data matrix of each olive sample. The mean values of each volatile compound (variables) obtained at 30-60-90 days of fermentation was analyzed and plotted. Bi-plots displaying PC1 vs. PC2 are illustrated in Figures 5, 6, which show the projection of the variables on the plane defined by the first and second principal components. The two planes made using the first two PCs showed clustering of the molecules into three groups. In all the four starter-driven fermentations, one group, consisting of aldehydes (hexanal in all the four cultivars; 2 methyl butanal, 3 methyl butanal in Leccino, Cellina di Nardò and Kalamàta; 2 methyl propanal in Leccino and Kalamàta; nonanal in Cellina di Nardò and Conservolea), were closely associated to the first fermentation stage (T30) (Figures 5, 6; Supplementary Tables 1–4). The second group consisted of higher alcohols (2-methyl-1-propanol, phenylethyl alcohol and 3-methyl-1-butanol in all the four fermentations; hexanol was present in Cellina di Nardò, Kalamàta and Conservolea; 3-methyl-1-butanol was present in Leccino, Kalamàta and Conservolea; propanol and heptanol were characteristics of Leccino), styrene and terpenes (3,7 dimethyl 1,3,7, octatriene in Cellina di Nardò, Kalamàta and Conservolea; limonene in Leccino; farnesene in Kalamàta and Conservolea; copaene in Conservolea; 2,6 dimethyl 2,4,6 octatriene and 3,7 dimethyl 1,6 octadien 3 ol in Kalamàta) associated in all fermentations with the middle stage of fermentation (T60) (Figures 5, 6; Supplementary Tables 1–4). third group contained acetate esters (isoamyl acetate, ethyl acetate) and esters (ethyl hexanoate, ethyl octanoate) in all the four fermentations. In addition, Greek cultivars were also characterized by the presence of ethyl lactate. All these compounds were linked to the final step in olive fermentation corresponding to the presence of inoculated LAB starter strains (T90) (Figures 5, 6; Supplementary Tables 1–4). All these compounds can be correlated to the metabolic activities of inoculated yeast starter strains.

### Radar plot

**Figure 7:**
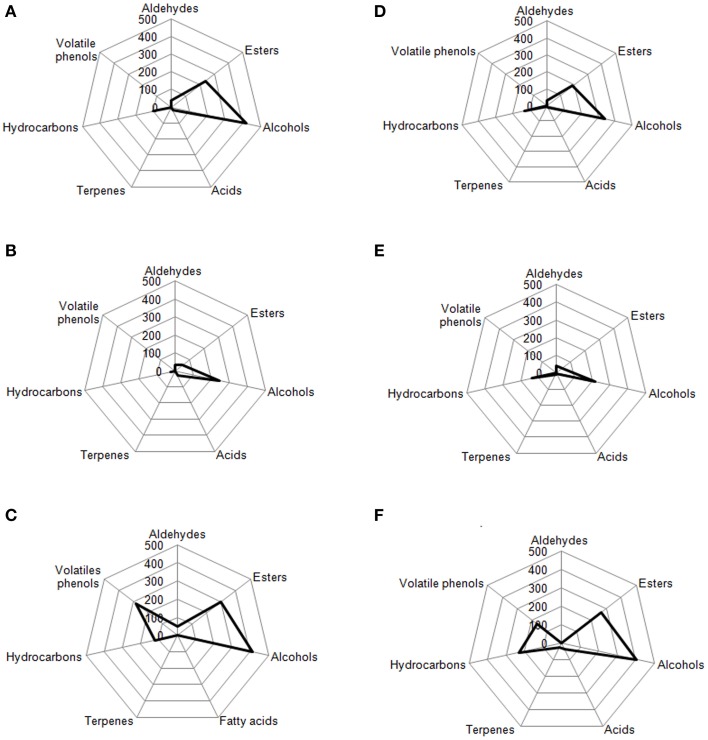
**Radar plot of all volatile classes associated to Leccino drupes (A) during fermentation driven by starters yeast and LAB, (B) during spontaneous fermentation process, (C) deriving from three different commercial products of Leccino cultivar**. Radar plot of all volatile classes associated to Cellina di Nardò drupes **(D)** during fermentation driven by starters yeast and LAB, **(E)** during spontaneous fermentation process, **(F)** deriving from three different commercial products of Cellina di Nardò cultivar.

**Figure 8:**
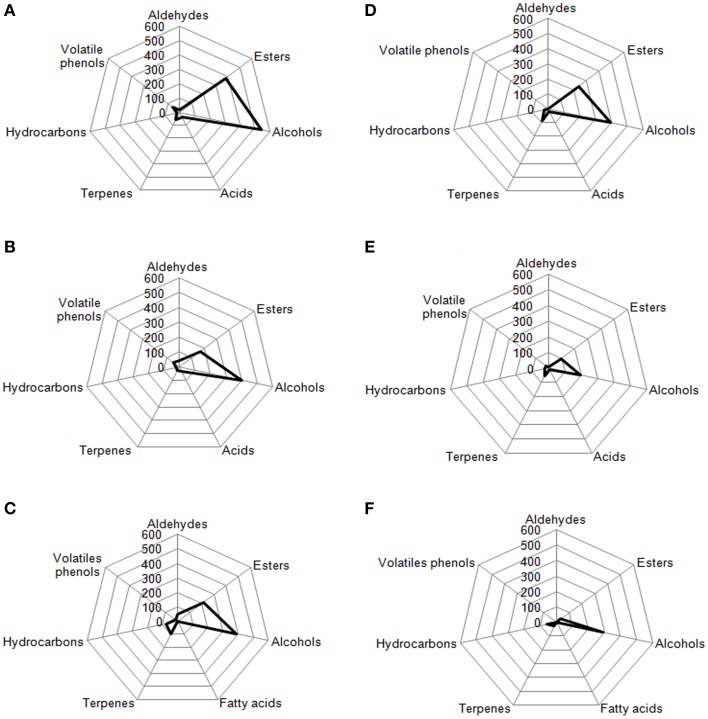
**Radar plot of volatiles classes associated to Kalamàta drupes (A) during fermentation driven by starters yeast and LAB, (B) during spontaneous fermentation process, (C) deriving from three different commercial products of Kalamàta cultivar**. Radar plot of volatiles classes associated to Conservolea drupes **(D)** during fermentation driven by starters yeast and LAB, **(E)** during spontaneous fermentation process, **(F)** deriving from three different commercial products of Conservolea cultivar.

In Figures 7, 8 are reported, for each analyzed table olive cultivar, the analytical radar plot of the final product obtained by starter-driven fermentation (Figures 7A,D, 8A,D), by the corresponding spontaneous fermentation (Figures 7B,E, 8B,E), by the mean values obtained by the analysis of three different table olive commercial products belonging to the same cultivar (Figures 7C,F, 8C,F). Using the information reported in literature, an attempt to associate volatile classes to expected odor descriptors has been produced: esters were associated to fruity, aldehydes to herbaceous, alcohols to winey-sweet, acids to acid, spicy, and terpenes to floral notes.

The volatile profile of Leccino and Cellina di Nardò table olives obtained by starter fermentation is compatible with the commercial characteristics of these two table olive cultivars, but with a significant reduction of volatile phenols (completely absent in Leccino and Cellina di Nardò produced by starters) and hydrocarbons (decreased of 7% in Leccino and of 13% Cellina di Nardò produced by starters). The use of starters enhanced the levels of volatile compounds in comparison with the corresponding spontaneous fermentation, improving esters, generally associated to fruity notes, higher alcohols associated to winey-sweet notes and maintaining molecules (C6-alcohols) characteristics of herbaceous notes. In Cellina di Nardò treated with starters esters increased of 2.8 fold, alcohols increased of 1.5 fold and acids increased of four fold. In Leccino starter-driven fermentations, esters increased of 4.8 fold, alcohols increased of 1.7 fold and acids decreased of 0.6 fold (Figure 7).

According to the obtained results, the volatile profiles of Kalamàta and Conservolea table olives were characterized by esters (fruity notes), higher alcohols (winey-sweet), C6-alcohols (herbaceous notes), and terpenes (floral attributes). In general, the treatment with starter microorganisms produced a mean content of esters and alcohol compounds greater than the corresponding natural fermentation process. In Kalamàta table olives produced by starters esters increased of 2.2 fold, alcohols increased of 1.4 fold, acids increased of 1.2 fold and terpenes increased of 2.4 fold. In Conservolea table olives produced by starters esters increased of 2.5 fold, alcohols increased of two fold, acids increased of 1.5 fold and terpenes increased of 0.9 fold (Figure 8). Starter-driven fermentations significantly improved volatile profile in comparison with the corresponding average sensorial representation obtained by three different commercial products for each cultivars. In both cultivars, table olives produced by starters showed an increase in esters of about two fold and alcohols of one fold. Also in this case, in starter-driven fermentations, levels of volatile phenols were maintained low and hydrocarbons concentration was significantly reduced (of about 75% in both cultivars).

## Discussion

In the present paper, starter formulations, composed of *S. cerevisiae/L. plantarum* for Leccino, *P. anomala*/*L. plantarum* for Cellina di Nardò, *S. cerevisiae* and *L. mesenteroides* for Kalamàta, *D. hansenii* and *L. plantarum* for Conservolea, previously selected by Bleve et al. (2014, 2015a), were validated as candidate starter in pilot-scale fermentations of two Italian (Leccino and Cellina di Nardò) and two Greek (Kalamàta and Conservolea) cultivars.

This new procedure for table olive fermentation allowed us to standardize the process, to reduce the time necessary to complete the process by a sequential inoculums strategy of a yeast strain and, after a optimal period of time, of a LAB strain. This method enables the process monitoring independent from empirical evaluation criteria and it, improves quality and safety of the final product.

As previously demonstrated by Bleve et al. (2014, 2015a) and Alves et al. (2012), yeasts are associated with the surface of Leccino, Cellina di Nardò, Kalamàta and Conservolea olive fruits and they play a substantial role throughout natural fermentations occurring during table olive production In fresh prepared brines of black olives, the growth of LAB is partially inhibited due to the presence of phenolic compounds. In addition, high level of NaCl during fermentation could affect the presence of LAB favoring the growth of yeasts. In fact, LAB remained undetectable until the last month, when they appeared and carried out lactic fermentation.

Several authors have hypothesized the importance of selection of yeasts as starter cultures during table olive processing (Silva et al., 2011; Arroyo-López et al., 2012b; Bevilacqua et al., 2012, 2013; Rodríguez-Gómez et al., 2012; Tofalo et al., 2013). Bevilacqua et al. (2013) selected, by a step-by-step procedure, 4 yeast strains belonging to *Kluyveromyces lactis, Wickerhamomyces anomalus*, and *Candida norvegica* intended as starters for Bella di Cerignola table olives. The authors hypothesized as a possible future perspective, the use of yeasts under factory conditions, alone or in presence of LAB for the formulation of mixed starter or as multifunctional starters.

Bonatsou et al. (2015) isolated yeast strains from Greek natural black table olive fermentations and selected most promising strains by applying a multivariate classification analysis.

Several recent studies suggested the importance of the presence of yeasts and LAB in table olive fermentations. Abbas (2006) demonstrated that the simultaneous presence of yeast and LAB favors the growth of LAB, Segovia Bravo et al. (2007) showed that in co-inoculation with *S. cerevisiae, L. pentosus* improved its ability to perform green olives fermentation, and in a similar manner, Tsapatsaris and Kotzekidou (2004), observed that the inoculum of *D. hansenii* 48 h before *L. plantarum*, enhanced its growth rate. Moreover, in Arbequina table olives fermentation, co-inoculation of *Candida diddensiae* and *L. pentosus* produced a reduction of *Enterobacteriacea*e, induced changes in yeast diversity with a sensitive improvement of the sensorial quality of olives (Hurtado et al., 2011). *C. boidinii* or *P. membranifaciens* have been also suggested as possible co-starter yeasts by Arroyo-López et al. (2008).

The molecular analyses performed on yeast and LAB populations isolated during the here performed fermentations revealed the imposition and dominance of our starters being these evidences consistent with those produced in previous similar studies (Argyri et al., 2014; Blana et al., 2014; Rodríguez-Gómez et al., 2014).

As expected, the oleuropein concentration decreased in the brines from starter-driven fermentations, since the demonstrated beta-glucosidase activity of our yeast and LAB strains (Bleve et al., 2014, 2015a), thus the olives resulting completely debittered. Moreover, hydroxytyrosol and tyrosol concentrations increased in all performed fermentations, probably due to the hydrolysis of oleuropein (Parinos et al., 2007; Ben Othman et al., 2009). The trends of sugars consumption and the corresponding synthesis of glycerol and ethanol confirmed the fermentation progress in all starter inoculated fermentations. Moreover, organic acids (lactic, citric, tartaric and acetic acid) were the more representive metabolites in the brines, according to data produced by spontaneous fermentations performed on green and black olive fermentations (Nychas et al., 2002; Chorianopoulos et al., 2005; Panagou et al., 2008; Bleve et al., 2014, 2015a). The use of starter ensured a decrease in pH value in all the four fermentations and the final value was maintained at about 4.0, which is satisfactory for naturally fermented black olive, as previously observed by Panagou et al. (2008) and Bleve et al. (2014, 2015a).

Ethanol concentration, very important for the organoleptic properties of naturally fermented black olives (Fleming et al., 1969), varied among the four table olive cultivars. Although these data revealed different yeasts activity and hetero-fermentative LAB, the ethanol content resulted similar in starter-driven and in the corresponding spontaneous fermentations. Microbial fermentation of glucose and fructose produces primary and secondary metabolites able to confer good organoleptic features and the typical flavor of the product. The presence of some fructophilic non-*Saccharomyces* yeasts commonly found in brine, together with varietal characteristics, could be responsible of the prevalence of residual glucose over fructose. The different content in lactic acid could be not related to total LAB count, but dependent from the quantity of fermentable sugars, the species and strains present during fermentation and their specific metabolic activities.

In this study, we validated the previously obtained evidences that specific molecules correlate with the microbial metabolism and that they can be suitable tools for the process monitoring (Bleve et al., 2014, 2015a). PCA analysis of the outcome of the chemical assays revealed that profiles of volatile molecules can be correlated with the growth of microorganisms. This analysis offers also the possibility to validate the use of volatile compounds suitable to be used as descriptors of each fermentation stage, as suggested by Bleve et al. (2014, 2015a). In all the four starter-driven fermentations, there is a first stage (30 days) characterized by a high aldehydes content, compounds characteristics of herbaceous flavors in fruits and vegetables. Higher alcohols, styrene and terpenes, compounds correlated with the metabolic activities of inoculated yeast starter strains, are associated to the second fermentation stage (60 days). These data confirmed that the yeasts are responsible of the first part of the process and suggest that these compounds can be indicative of yeast metabolism (Swiegers and Pretorius, 2005). Prevalent alcohols were 2+3 methyl-1-butanol and phenylethylalcohol (isoamyl alcohol, fruity-winey notes), hexanol (fruity-green notes) and cis 3-hexen-1-ol (green notes), previously observed in spontaneous fermentation of the four table olive cultivars (Bleve et al., 2014, 2015a). During the third fermentation stage (90 days), table olive fermented by mixed starters mainly contained acetate esters (isoamyl acetate, ethyl acetate), esters (ethyl hexanoate and ethyl octanoate) and acids, probably due to the different pathway undertaken by LAB enzymes.

It is very important to note that the use of starter microorganisms allowed to significantly reduce the time of fermentation process from 180 to 90 days. Moreover, the first stage of fermentation shifted from 90 (spontaneous fermentation) to 60 days (starter-driven fermentation) and the second step shifted from 180 (spontaneous fermentation) to 90 days (starter-driven fermentation).

In this work the analysis, carried out by GC–MS on the four different black table olive cultivars, helped us in developing a volatile radar plot as a picture of the entire process evolution. In all studied cases, the use of sequential inoculation strategy of selected autochthonous yeast and LAB starter strains produced a volatile profile more rich in compounds that can be associated to fruit, winey-sweet and herbaceous and floral attributes than the corresponding spontaneous fermentation. The treatment with starter microorganisms produced a final product with optimal organoleptic characteristics and with a significant reduction of volatile phenols and hydrocarbons.

The proposed method based on volatile radar plot could be suggested to chemical analysts to produce a preliminary representation of the aroma fingerprint of table olive cultivar by linking the chemical information to expected odor notes. Chemical analyses used in this paper are not to be considered alternative to official IOC protocol for sensory evaluation. In fact, future analyses will be performed involving trained panel experts for table olives tasting and for analyses of objective sensorial descriptors in these products.

For the first time, yeast and LAB isolates were used as autochthonous starters for pilot-scale production of Leccino, Cellina di Nardò, Conservolea and Kalamàta olive fermented table olives. A sequential inoculation strategy, consisting in firstly inoculating selected yeast strains and then inoculating selected LAB strains, was validated as a new approach in controlling the table olive fermentation process. Sequential starters were able to dominate the fermentations and their use allowed to shorten the time of fermentation (90 days), to standardize the process and to improve organoleptic properties of the above described four cultivars of olives, in comparison with corresponding commercial products. The here described method for table olive production has been deposited as Italian patent MI 2013A002063 and has been applied for European patent Nr. 14197402.2. (Bleve et al., 2013, 2015b).

Our future challenge will be to investigate these strains for their probiotic characteristics, in order to produce functional olives. In fact, several studies demonstrated that the use of LAB as starter for table olive production can produce beneficial effects for human health (Lavermicocca et al., 2005; De Bellis et al., 2010; Silva et al., 2011; Argyri et al., 2013; Bautista-Gallego et al., 2013; Blana et al., 2014). Interpreting the new frontier in starter research (Bevilacqua et al., 2013), also yeast strains will be evaluated for their probiotic properties. Large-scale experiments are now in progress in two table olive producing factories in Apulia (placeItaly), to definitely validate the use of these autochthonous yeast and LAB strains s starter cultures for large-scale productions.

## Author contributions

Fundamental contributions to the conception and design of the work, acquisition, analysis and interpretation of data (GB, MT, GM, EP, FG, LT); acquisition, analysis, elaboration and interpretation of data (MT, GB, MD, FR, LT, VF); drafting the work and revising it critically for intellectual content (GB, FG, GM, MT-M, AL). All authors approved the final version of the manuscript to be submitted for publication and agreed to be accountable for all aspects of the work in ensuring that questions related to the accuracy and integrity of any part of the work are appropriately investigated and resolved.

### Conflict of interest statement

The authors declare that the research was conducted in the absence of any commercial or financial relationships that could be construed as a potential conflict of interest.
