# Supplementary material for: New process for production of fermented black table olives using selected autochthonous microbial resources
Source: Front Microbiol. 2015 Sep 24;6:1007. doi: 10.3389/fmicb.2015.01007 (PMC4585182; doi:10.3389/fmicb.2015.01007)
Supplement: Supplementary file 1 [file Table1.DOCX]

***Supplementary Material***

**New process for production of fermented black table olives using selected autochthonous microbial resources**

Running title: Starters for black table olives

Maria Tufariello^a^, Francesca Anna Ramires^a^, Miriana Durante^a^, Francesco Grieco^a^, Luca Tommasi^b^, Ezio Perbellini^c^, Vittorio Falco^a^, Maria Tasioula-Margari^d^, Antonio Francesco Logrieco^e^, Giovanni Mita^a^ and Gianluca Bleve^a *^

^a^ Consiglio Nazionale delle Ricerche - Istituto di Scienze delle Produzioni Alimentari, Unità Operativa di Lecce, Lecce, Italy

^b^ Associazione “Olivicoltori di Puglia”, Lecce, Italy

^c^ Agricola Nuova Generazione Soc. Coop., Martano (LE), Italy

^d^ Department of Chemistry, Section of Food Chemistry, University of Ioannina, Ioannina, Greece

^e^ Consiglio Nazionale delle Ricerche - Istituto di Scienze delle Produzioni Alimentari, Bari, Italy

^*^ Correspondence:

Dr. Gianluca Bleve

Istituto di Scienze delle Produzioni Alimentari

Consiglio Nazionale delle Ricerche

Unità Operativa di Lecce

Via Provinciale Lecce-Monteroni

73100 Lecce, Italy

gianluca.bleve@ispa.cnr.it

1. **Supplementary Figures and Tables**

## Supplementary Tables

**Supplementary Table 1.** SPME/GC–MS quantitative data, including concentration (µg/kg) with standard deviation (SD) of all the volatile compounds identified in Leccino table olives.

|  | Starter-driven fermentation | | |  | Natural fermentation | | |
| --- | --- | --- | --- | --- | --- | --- | --- |
| ***Compounds*** | 30 days | 60 days | 90 days |  | 30 days | 60 days | 90 days |
|  | mean ± SD μg/kg* | mean ± SD ug/kg* | mean ± SD ug/kg* |  | mean ± SD μg/kg* | mean ± SD ug/kg* | mean ± SD ug/kg* |
| **Aldehydes** |  |  |  |  |  |  |  |
| 2 Methyl propanal | 7.78 a±0.62 | Nd | Nd |  | Nd | 8.81 c±0.21 | 3.64 b±0.11 |
| 2 Methyl butanal | 19.32 c±0.21 | 12.54 b±3.96 | 8.92 a±1.10 |  | 23.47 b±2.03 | 23.78 b±1.81 | 12.35 a ±1.05 |
| 3 Methyl butanal | 12.28 c±2.65 | 10.21 b±2.15 | 8.49 a±2.15 |  | 33.01 c±3.11 | 24.69 b±2.56 | 17.72 a±2.03 |
| Hexanal | 5.56 b±0.33 | 3.78 a±0.23 | Nd |  | 3.94 b±0.22 | Nd | Nd |
| Nonanale | Nd | Nd | Nd |  | 13.75 b±1.12 | Nd | Nd |
| Benzaldehyde | 5.45 a±0.54 | 15.36 b±3.75 | 19.87 c±5.82 |  | 2.28 b±0.12 | Nd | Nd |
| *Total amounts* | **50.39±4.34** | **41.9±10.10** | **37.28±9.07** |  | **76.44±6.60** | **57.28±4.58** | **33.71±3.19** |
| **Esters** |  |  |  |  |  |  |  |
| Methyl acetate | Nd | 33.36 b±5.29 | 22.58 a±0.55 |  | Nd | 6.04 b±2.30 | Nd |
| Ethyl acetate | 3.90 a±0.14 | 96.04 b±7.48 | 120.06 c±6.60 |  | 8.53 a±1.11 | 24.79 b±4.75 | 36.11 c±2.34 |
| 2 Methyl ethyl butanoate | Nd | Nd | 2.31 a±0.26 |  | Nd | 3.25 a±0.06 | Nd |
| 3 Methyl ethyl butanoate | Nd | Nd | Nd |  | Nd | 3.21 a±0.08 | Nd |
| Isoamyl acetate | 3.08 a ±0.03 | 12.00 b±3.46 | 29.49 c±2.66 |  | 2.66 a±0.14 | Nd | Nd |
| Ethyl hexanoate | 11.07 a±0.87 | 15.25 b±1.49 | 24.17 c±0.41 |  | 8.53 b±0.55 | Nd | 6.17a±0.62 |
| Ethyloctanoate | 12.05 a±1.28 | 22.02 b±3.72 | 30.36 c±1.03 |  | Nd | Nd | 7.34 b±0.25 |
| Hexyl acetate | Nd | Nd | 3.46 a±0.09 |  | Nd | Nd | Nd |
| Ethyl lactate | Nd | Nd | 3.73 a±0.01 |  | Nd | Nd | Nd |
| *Total amounts* | **30.11±2.31** | **179.65±21.44** | **236.15±11.61** |  | **19.72±2.37** | **37.3±2.45** | **49.61±3.21** |
| **Alcohols** |  |  |  |  |  |  |  |
| Ethanol | 75.74 a±6.96 | 174.91 b±8.58 | 200.51 c±10.64 |  | 28.43 a±3.11 | 164.74 b±8.36 | 178.26 c±8.21 |
| Propanol | Nd | 5.32 a±0.53 | Nd |  | Nd | Nd | Nd |
| 2 Methyl propanol | Nd | 31.25 a±6.11 | 62.88 b±2.36 |  | Nd | Nd | Nd |
| 3 Methyl butanol | 29.73 a±3.90 | 80.53 b±9.63 | 103.62 c±6.80 |  | 28.01 a±3.11 | 68.89 c±9.82 | 49.06 b±4.25 |
| Hexanol | 9.69 a±1.47 | 22.93 b±3.30 | 23.34 b±4.77 |  | 7.30 a±0.54 | 13.97 b±1.73 | 7.23 a±0.56 |
| Hexen-3-ol (z) | Nd | 6.89 a±0.98 | 7.78 a±0.20 |  | Nd | 1.92 a±0.07 | Nd |
| Heptanol | 2.12 a±0.08 | 6.61 b±1.38 | Nd |  | Nd | Nd | Nd |
| Benzylalcohol | 3.94 a ±1 | 16.12 c±1.83 | 13.24 b±1.74 |  | 4.00 b±0.12 | Nd | 2.97a±0.36 |
| Phenylethylalcohol | Nd | 6.60 b±1.17 | 9.50 c±0.43 |  | 12.74 b±1.03 | 14.69 b±4.34 | 10.11 a±3.05 |
| *Total amounts* | **121.22±13.41** | **351.16±33.51** | **420.87±26.94** |  | **80.48±7.91** | **264.21±24.32** | **247.63±16.43** |
| **Acids** |  |  |  |  |  |  |  |
| Acetic acid | 9.88 a±2.60 | 11.22 b±2.37 | 15.18 c±4.20 |  | 11.18a±1.12 | 15.62 b±4.05 | 31.67 c±4.43 |
| 3 Methyl butanoic acid | Nd | Nd | 3.62a±0.16 |  |  |  |  |
| *Total amounts* | **9.88±2.60** | **11.23±2.37** | **18.79±4.36** |  | **11.18a±1.12** | **15.62 b±4.05** | **31.67 c±4.43** |
| **Terpenes** |  |  |  |  |  |  |  |
| Limonene | Nd | 8.60 a±2.11 | Nd |  |  |  |  |
| *Total amounts* |  | **8.60 a±2.11** | Nd |  |  |  |  |
| **Hydrocarbons** |  |  |  |  |  |  |  |
| Octane | 11.06 a ±2.40 | 18.32b±2.10 | 23.21 b±3.64 |  | 17.45 a±4.34 | 16.94 a±4.23 | 18.11a±2.76 |
| Styrene | 12.52 a ±4.71 | 26.83b±2.34 | 38.84 c±9.35 |  | 15.94 a±3.26 | 22.57 c±4.87 | 27.28 b±4.05 |
| *Total amounts* | **33.58±7.11** | **45.15±4.44** | **62.05±12.99** |  | **33.4±7.60** | **39.51±9.10** | **27.28±6.81** |

^*^ Results expressed as mean± standard deviation (SD); ND: not detected.

For each compound, according to the result of the one-way ANOVA test, concentration values that do not share a common superscript are significantly different (P < 0.05). a,b,c: the different letters indicate significant differences among stage of fermentation in the same volatile classes (p<0.05).
