# Supplementary material for: New process for production of fermented black table olives using selected autochthonous microbial resources
Source: Front Microbiol. 2015 Sep 24;6:1007. doi: 10.3389/fmicb.2015.01007 (PMC4585182; doi:10.3389/fmicb.2015.01007)
Supplement: Supplementary file 2 [file Table2.DOCX]

***Supplementary Material***

**New process for production of fermented black table olives using selected autochthonous microbial resources**

Running title: Starters for black table olives

Maria Tufariello^a^, Francesca Anna Ramires^a^, Miriana Durante^a^, Francesco Grieco^a^, Luca Tommasi^b^, Ezio Perbellini^c^, Vittorio Falco^a^, Maria Tasioula-Margari^d^, Antonio Francesco Logrieco^e^, Giovanni Mita^a^ and Gianluca Bleve^a *^

^a^ Consiglio Nazionale delle Ricerche - Istituto di Scienze delle Produzioni Alimentari, Unità Operativa di Lecce, Lecce, Italy

^b^ Associazione “Olivicoltori di Puglia”, Lecce, Italy

^c^ Agricola Nuova Generazione Soc. Coop., Martano (LE), Italy

^d^ Department of Chemistry, Section of Food Chemistry, University of Ioannina, Ioannina, Greece

^e^ Consiglio Nazionale delle Ricerche - Istituto di Scienze delle Produzioni Alimentari, Bari, Italy

^*^ Correspondence:

Dr. Gianluca Bleve

Istituto di Scienze delle Produzioni Alimentari

Consiglio Nazionale delle Ricerche

Unità Operativa di Lecce

Via Provinciale Lecce-Monteroni

73100 Lecce, Italy

gianluca.bleve@ispa.cnr.it

1. **Supplementary Figures and Tables**

## Supplementary Tables

**Supplementary Table 2.** SPME/GC–MS quantitative data, including concentration (µg/kg) with standard deviation (SD) of all the volatile compounds identified in Cellina di Nardò table olives.

|  |  |  |  |  |  | |  | |  | |
| --- | --- | --- | --- | --- | --- | --- | --- | --- | --- | --- |
|  | Starter-driven fermentation | | |  | Natural fermentation | | |  | |  |
| ***Compounds*** | 30 days | 60 days | 90 days |  | 30 days | 60 days | | | 90 days | |
|  | mean ± SD μg/kg* | mean ± SD ug/kg* | mean ± SD ug/kg* |  | mean ± SD μg/kg* | mean ± SD ug/kg* | | | mean ± SD ug/kg* | |
| **Aldehydes** |  |  |  |  |  | |  | |  | |
| 2 Methyl butanal | 7.05b±1.74 | 5.28a±0.55 | 5.02a±0.44 |  | 5.32b±0.66 | | 5.10b±0.54 | | 2.80a±0.34 | |
| 3 Methyl butanal | 8.01b±2.04 | 4.77a±0.67 | 4.10a±0.54 |  | 5.44b±0.36 | | 5.03b±0.56 | | 2.20a±0.43 | |
| Hexanal | 7.58c±2.76 | 5.77b±0.87 | 4.05a±0.33 |  | 3.76a±0.76 | | 5.80b±0.87 | | 9.44c±2.05 | |
| Heptanal | Nd | Nd | Nd |  | 2.43a±0.06 | | Nd | | 5.58b±1.11 | |
| Nonanale | 74.06c±7.04 | 59.15b±8.65 | 18.89a±4.65 |  | 28.66b±5.11 | | 12.02a±4.05 | | 11.68a±2.78 | |
| 2 Decenale | 17.34b±3.05 | 13.23a±2.11 | Nd |  | Nd | | Nd | | Nd | |
| *Total amounts* | **114.04±16.63** | **88.20±12.85** | **32.07±5.96** |  | **45.61±6.96** | | **27.95±6.02** | | **31.70±6.71** | |
| **Esters** |  |  |  |  |  | |  | |  | |
| Ethyl acetate | 7.25a±2.11 | 20.50b±3.06 | 40.50c±4.04 |  | 6.89a±1.04 | | 23.34b±3.48 | | 5.74a±0.76 | |
| Isoamyl acetate | 4.90a±0.34 | 27.87b±2.54 | 46.40c±5.11 |  | Nd | | 5.05a±0.88 | | 5.46a±0.54 | |
| Ethyl hexanoate | 3.87a±0.65 | 31.10b±5.76 | 54.53c±3.74 |  | Nd | | 4.86a±0.43 | | 18.30b±4.34 | |
| 3 Hexen ol acetate (z) | Nd | 11.16a±2.05 | 33.63b±4.38 |  | Nd | | Nd | | 2.80±0.22 | |
| Ethyl octanoate | Nd | 5.32a±0.66 | 12.81b±3.04 |  | Nd | | Nd | | 9.37±2.05 | |
| *Total amounts* | **16.02±3.10** | **95.95±14.07** | **187.87±20.31** |  | **6.89±1.04** | | **33.25±4.79** | | **41.67±7.91** | |
| **Alcohols** |  |  |  |  |  | |  | |  | |
| Ethanol | 100.20a±11.65 | 106.70a±11.32 | 170.60b±12.11 |  | 48.57a±12.04 | | 137.02c±11.43 | | 94.25b±7.11 | |
| 2 Methyl propanol | 5.02a±0.67 | 4.84a±0.65 | Nd |  | 6.72a±0.56 | | 10.85b±1.05 | | Nd | |
| 3 Methyl butanol | 39.40a±8.04 | 63.07c±8.11 | 52.54b±5.06 |  | 47.82b±7.04 | | 62.22c±7.12 | | 34.48a±5.05 | |
| Hexanol | 20.15a±3.11 | 27.89b±3.05 | 28.77b±4.56 |  | 29.47b±4.12 | | 27.05b±3.55 | | 22.91a±4.65 | |
| Hexen-3-ol (z) | 19.74a±4.05 | 20.81a±4.04 | 31.07b±5.76 |  | 22.27a±3.55 | | 22.16a±4.07 | | 19.99a±3.36 | |
| Heptanol | 5.40a±0.76 | 14.63b±3.63 | 20.97c±3.06 |  | 5.42a±0.54 | | 6.17a±1.45 | | 17.1b±2.98 | |
| Phenylethylalcohol | 18.21a±3.06 | 30.47b±3.88 | Nd |  | Nd | | Nd | | Nd | |
| 1 Nonanolo | Nd | 10.53a±1.65 | 36.47b±4.11 |  | Nd | | 3.10±0.75 | | 29.73±4.05 | |
| *Total amounts* | **208.12±31.34** | **278.93±36.33** | **340.43±34.66** |  | **160.27±27.85** | | **268.56±29.42** | | **218.46±27.20** | |
| **Acids** |  |  |  |  |  | |  | |  | |
| Acetic acid | Nd | Nd | 8.61±1.44 |  | Nd | | Nd | | 2.16±0.05 | |
| *Total amounts* |  |  | **8.61±1.44** |  |  | |  | | **2.16±0.05** | |
| **Terpenes** |  |  |  |  |  | |  | |  | |
| 3,7 Dimethyl 1,3,7 octatriene | Nd | 3.38a±0.06 | 3.78a±0.34 |  | Nd | | 0.22a±0.05 | | 6.74b±1.04 | |
| *Total amounts* |  | **3.38±0.06** | **3.78±0.34** |  |  | | **0.22±0.05** | | **6.74±1.04** | |
| **Hydrocarbons** |  |  |  |  |  | |  | |  | |
| Octane | 12.26a±3.11 | 26.40b±5.05 | 34.80c±5.65 |  | 38.88b±4.32 | | 31.24a±3.75 | | 58.80c±4.33 | |
| Styrene | 20.99a±6.04 | 23.72a±7.04 | 35.27b±6.12 |  | 27.73a±3.03 | | 70.8b±5.22 | | 76.84b±6.43 | |
| *Total amounts* | **33.25±9.15** | **50.12±12.09** | **70.07±11.77** |  | **66.61±7.35** | | **102.04±8.97** | | **135.64±10.76** | |
