# Supplementary material for: New process for production of fermented black table olives using selected autochthonous microbial resources
Source: Front Microbiol. 2015 Sep 24;6:1007. doi: 10.3389/fmicb.2015.01007 (PMC4585182; doi:10.3389/fmicb.2015.01007)
Supplement: Supplementary file 3 [file Table3.DOCX]

***Supplementary Material***

**New process for production of fermented black table olives using selected autochthonous microbial resources**

Running title: Starters for black table olives

Maria Tufariello^a^, Francesca Anna Ramires^a^, Miriana Durante^a^, Francesco Grieco^a^, Luca Tommasi^b^, Ezio Perbellini^c^, Vittorio Falco^a^, Maria Tasioula-Margari^d^, Antonio Francesco Logrieco^e^, Giovanni Mita^a^ and Gianluca Bleve^a *^

^a^ Consiglio Nazionale delle Ricerche - Istituto di Scienze delle Produzioni Alimentari, Unità Operativa di Lecce, Lecce, Italy

^b^ Associazione “Olivicoltori di Puglia”, Lecce, Italy

^c^ Agricola Nuova Generazione Soc. Coop., Martano (LE), Italy

^d^ Department of Chemistry, Section of Food Chemistry, University of Ioannina, Ioannina, Greece

^e^ Consiglio Nazionale delle Ricerche - Istituto di Scienze delle Produzioni Alimentari, Bari, Italy

^*^ Correspondence:

Dr. Gianluca Bleve

Istituto di Scienze delle Produzioni Alimentari

Consiglio Nazionale delle Ricerche

Unità Operativa di Lecce

Via Provinciale Lecce-Monteroni

73100 Lecce, Italy

gianluca.bleve@ispa.cnr.it

1. **Supplementary Figures and Tables**

## Supplementary Tables

**Supplementary Table 3.** SPME/GC–MS quantitative data, including concentration (µg/kg) with standard deviation (SD) of all the volatile compounds identified in Kalamata table olives.

|  | Starter-driven fermentation | | |  | Natural fermentation |  |  |
| --- | --- | --- | --- | --- | --- | --- | --- |
| ***Compounds*** | 30 days | 60 days | 90 days |  | 30 days | 60 days | 90 days |
|  | mean ± SD μg/kg* | mean ± SD ug/kg* | mean ± SD ug/kg* |  | mean ± SD μg/kg* | mean ± SD ug/kg* | mean ± SD ug/kg* |
| **Aldehydes** |  |  |  |  |  |  |  |
| 2 Methyl propanal | 8.50 a±0.22 | Nd | Nd |  | 40.74 a±4.89 | Nd | Nd |
| 2 Methyl butanal | 7.89 c±0.22 | 5.77 b±0.66 | 4.67 a±1.02 |  | 48.14 b±3.32 | 12.44 a±3.47 | 13.43 a±2.05 |
| 3 Methyl butanal | 12.81 c±0.34 | 6.10a±0.42 | 7.41 b±0.35 |  | 27.65 c±5.54 | 18.93 b±3.63 | 12.93 a±2.99 |
| Hexanal | 16.24 b±2.11 | 5.77 a±0.67 | 5.42 a±0.50 |  | 8.59 a±1.31 | 12.27 c±2.78 | 10.66 b±1.90 |
| Nonanal | Nd | 12.2±1.14 | Nd |  | Nd | 8.06 a±0.47 | Nd |
| Benzaldehyde | Nd | Nd | Nd |  | Nd | 6.55 b±1.84 | 4.70 a±0.30 |
| *Total amounts* | **45.44±2.89** | **29.84±2.89** | **17.50±0.85** |  | **125.12±14.07** | **58.25±6.19** | **41.72±7.23** |
| **Esters** |  |  |  |  |  |  |  |
| Methyl acetate | Nd | 7.92 a±0.58 | 47.94 b±4.02 |  | Nd | 18.20 a±5.59 | 22.23 b±4.25 |
| Ethyl acetate | 35.61 a±7.05 | 69.33 b±5.60 | 104.93 c±7.45 |  | 26.32 a±3.11 | 36.70 b±6.48 | 69.80 c±1.12 |
| Ethyl propionate | Nd | Nd | 8.37 a±1.52 |  | Nd | Nd | 9.80 a |
| Propyl acetate | Nd | Nd | Nd |  | Nd | Nd | 8.82 a |
| 2 Methyl ethyl butanoate | Nd | 12.39 a±3.06 | nd |  | Nd | Nd | nd |
| Isoamyl acetate | Nd | 71.62 a±6.66 | 96.67 b±8.41 |  | 14.54 b±4.32 | 16.43 c±4.14 | 9.05 a±0.44 |
| Ethyl hexanoate | Nd | Nd | 29.48 a±4.17 |  | Nd | Nd | 19.76 a±3.41 |
| 3 Hexen ol acetate | Nd | 5.13 a±0.24 | nd |  | Nd | 2.13 a±0.11 | Nd |
| Ethyl lactate | Nd | 20.11 a±4.31 | 61.51 b±8.18 |  | Nd | 4.04 a±0.40 | 8.67 b±2.03 |
| Ethyl octanoate | Nd | 25.40 a±4.08 | 29.06 b±3.68 |  | Nd | 18.30 a±4.61 | 20.32 a±5.05 |
| *Total amounts* | **35.61±7.05** | **211.90±24.53** | **377.95±41.40** |  | **40.86±7.43** | **95.80±21.33** | **168.45±17.30** |
| **Alcohols** |  |  |  |  |  |  |  |
| Ethanol | 123.74 a±4.92 | 315.32 b±7.33 | 349.64 c±8.12 |  | 63.27a ±5.03 | 119.71 b±7.54 | 232.92 b±8.85 |
| 2 Butanol | Nd | Nd | 16.41 a±3.93 |  | Nd | Nd | Nd |
| 1 Propanol | Nd | Nd | 8.71 a±2.32 |  | Nd | 11.67 b±3.35 | 4.13 a±0.75 |
| 2 Methyl propanol | 11.24 a±3.04 | 42.15 c±4.11 | 31.03 b±5.15 |  | Nd | 4.02 a±0.22 | 10.45 b±2.45 |
| 3 Methyl butanol | 22.10 a±2.81 | 93.40 c±7.42 | 64.13 b±5.36 |  | 34.42 a±4.08 | 43.46 b±5.80 | 76.54 c±9.34 |
| Hexanol | 7.31 a±0.65 | 35.93 c±5.21 | 18.58 b±5.65 |  | 12.70 a±2.65 | 14.42 b±2.57 | 11.65 a±1.29 |
| 3 Hexen ol (Z) | 48.03c±4.32 | 23.80 b±4.01 | 10.19 a±3.42 |  | 38.75 c±3.80 | 26.86 b±4.11 | 22.78 a±1.57 |
| Benzylalcohol | Nd | 9.32 a±0.73 | 14.9 b±2.5 |  | Nd | 16.24 a±1.21 | 16.92 a±3.76 |
| Phenylethyl alcohol | Nd | 32.48 b±4.20 | 28.53 a±3.62 |  | Nd | 21.20 b±2.16 | 16.79 a±4.08 |
| *Total amounts* | **212.43±15.74** | **552.4±33.01** | **542.12±40.07** |  | **149.14±15.56** | **257.58±26.96** | **399.18±32.09** |
| **Acids** |  |  |  |  |  |  |  |
| Acetic acid | Nd | 25.89 a±5.13 | 39.0 b±6.72 |  | 6.26 a±0.32 | 9.30 b±2.74 | 32.62 c±4.56 |
| **Terpenes** |  |  |  |  |  |  |  |
| α-Farnesene | 16.87 b±3.05 | Nd | 5.16 a±0.54 |  | Nd | Nd | 7.05 a±2.23 |
| 3,7 Dimethyl 1,3,7 octatriene | 75.56 c±9.76 | 13.79 b±2.22 | 10.13 a±2.15 |  | 8.49±2.31 | 11.98 b±3.06 | 14.04 c±2.35 |
| 2,6 Dimethyl 2,4,6 octatriene | Nd | 52.68 b±4.75 | 42.29 a±7.63 |  | 12.51 a±4.11 | 12.22 a±5.11 | Nd |
| 3,7 Dimethyl 1,6 octadien 3 ol | Nd | 5.24 b±0.67 | 3.86 a±0.43 |  | Nd | 4.36 a±1.32 | 4.90 a±0.50 |
| *Total amounts* | **92.43±12.81** | **57.92±7.64** | **61.45±10.75** |  | **21.00±6.42** | **28.56±9.49** | **25.99±4.58** |
| **Volatiles phenols** |  |  |  |  |  |  |  |
| Guaiacol | Nd | 4.77a±0.37 | 5.29 a |  | Nd | 4.39 a±1.23 | 5.18 a±0.80 |
| Methyl guaiacol | Nd | 7.39 a±1.75 | 55.18 b |  | Nd | 14.93 a±3.80 | 46.74 b±5.63 |
| *Total amounts* |  | **12.16±2.12** | **60.47±6.97** |  |  | **19.32±5.03** | **51.92±6.43** |
| **Hydrocarbons** |  |  |  |  |  |  |  |
| Octane | 26.30 b±4.11 | 6.56 a±1.23 | Nd |  | 37.89 b±4.80 | 11.43 a±2.71 | Nd |
| Styrene | 14.15 a±4.62 | 27.35 b±5.61 | 23.81 ab±4.86 |  | 50.01 b±5.74 | 51.87 b±4.64 | 19.07 a±3.04 |
| *Total amounts* | **40.45±8.72** | **33.91±6.84** | **23.81±4.86** |  | **87.90±10.54** | **63.30±7.35** | **19.07±3.04** |
