# Supplementary material for: New process for production of fermented black table olives using selected autochthonous microbial resources
Source: Front Microbiol. 2015 Sep 24;6:1007. doi: 10.3389/fmicb.2015.01007 (PMC4585182; doi:10.3389/fmicb.2015.01007)
Supplement: Supplementary file 4 [file Table4.DOCX]

***Supplementary Material***

**New process for production of fermented black table olives using selected autochthonous microbial resources**

Running title: Starters for black table olives

Maria Tufariello^a^, Francesca Anna Ramires^a^, Miriana Durante^a^, Francesco Grieco^a^, Luca Tommasi^b^, Ezio Perbellini^c^, Vittorio Falco^a^, Maria Tasioula-Margari^d^, Antonio Francesco Logrieco^e^, Giovanni Mita^a^ and Gianluca Bleve^a *^

^a^ Consiglio Nazionale delle Ricerche - Istituto di Scienze delle Produzioni Alimentari, Unità Operativa di Lecce, Lecce, Italy

^b^ Associazione “Olivicoltori di Puglia”, Lecce, Italy

^c^ Agricola Nuova Generazione Soc. Coop., Martano (LE), Italy

^d^ Department of Chemistry, Section of Food Chemistry, University of Ioannina, Ioannina, Greece

^e^ Consiglio Nazionale delle Ricerche - Istituto di Scienze delle Produzioni Alimentari, Bari, Italy

^*^ Correspondence:

Dr. Gianluca Bleve

Istituto di Scienze delle Produzioni Alimentari

Consiglio Nazionale delle Ricerche

Unità Operativa di Lecce

Via Provinciale Lecce-Monteroni

73100 Lecce, Italy

gianluca.bleve@ispa.cnr.it

1. **Supplementary Figures and Tables**

## Supplementary Tables

**Supplementary Table 4.** SPME/GC–MS quantitative data, including concentration (µg/kg) with standard deviation (SD) of all the volatile compounds identified in Conservolea table olives.

|  |  | Starter-driven fermentation |  |  | Natural fermentation |  |  |
| --- | --- | --- | --- | --- | --- | --- | --- |
| ***Compounds*** | 30 days | 60 days | 90 days |  | 30 days | 60 days | 90 days |
|  | mean ± SD μg/kg* | mean ± SD ug/kg* | mean ± SD ug/kg* |  | mean ± SD μg/kg* | mean ± SD ug/kg* | mean ± SD ug/kg* |
| **Aldehydes** |  |  |  |  |  |  |  |
| Hexanal | 4.51b±0.88 | 3.53a±0.51 | Nd |  | 5.78 b±0.56 | 3.50 a±0.63 | Nd |
| Nonanal | 8.49 b±2.21 | 5.81 a±0.22 | Nd |  | Nd | 7.95 a±0.74 | Nd |
| 2 Decenal | 7.50 b±1.60 | Nd | 2.14 a±0.33 |  | 6.81 a±1.11 | Nd | 7.79 b±1.22 |
| Benzaldehyde | 20.50 b±4.69 | 2.90 a±0.11 | Nd |  | Nd | Nd | Nd |
| *Total amounts* | **41.00±9.38** | **12.24±0.84** | **2.14±0.33** |  | **12.58±1.67** | **11.45±1.38** | **7.79±1.22** |
| **Esters** |  |  |  |  |  |  |  |
| Ethyl acetate | 17.16 a±2.04 | 59.38 b±4.73 | 72.06 c±6.82 |  | 13.78 a±4.11 | 35.69 b±4.16 | 38.57 b±2.10 |
| Ethyl propanoate | Nd | Nd | 6.90 a±0.23 |  | Nd | Nd | 5.87 a±0.84 |
| Propyl acetate | Nd | Nd | Nd |  | Nd | Nd | 4.94 a±0.45 |
| Acetic acid methylpropyl ester | Nd | Nd | Nd |  | Nd | Nd | 4.66 a±0.54 |
| 2 Methyl ethyl butanoate | Nd | Nd | 0.83 a±0.07 |  | Nd | Nd | 1.47 a±0.32 |
| 3 Methyl ethyl butanoate | Nd | Nd | 1.32 a±0.04 |  | Nd | Nd | 1.80 a±0.25 |
| Isoamyl acetate | 21.66 a±3.04 | 68.12 b±4.40 | 92.54 c±7.78 |  | Nd | 13.09 a±1.46 | 14.82 a±3.06 |
| Ethyl hexanoate | Nd | Nd | 10.29 a±2.24 |  | Nd | Nd | Nd |
| Hexyl acetate | Nd | 4.95 a±0.22 | 9.38 b±1.75 |  | Nd | 4.15a±0.62 | 5.11a±0.62 |
| Hexen-3-ol (z) acetate | Nd | Nd | 1.42 a±0.04 |  | Nd | 0.97 a±0.05 | 1.24 a±0.05 |
| Ethyl lactate | Nd | 13.34 a±1.91 | 40.04 b±4.04 |  | Nd | 3.04 a±0.05 | 9.67 b±2.30 |
| Ethyl octanoate | Nd | 4.25 a±0.54 | 8.12 b±1.40 |  | Nd | 9.08 b±1.65 | 7.57 a±0.72 |
| Methyl salicylate |  |  |  |  | Nd | 3.35 a±0.07 | 2.59 a±0.08 |
| *Total amounts* | **38.82** | **150.04±11.80** | **242.90±24.57** |  | **13.78±4.11** | **69.38±8.06** | **98.31±11.33** |
| **Alcohols** |  |  |  |  |  |  |  |
| Ethanol | 36.58 a±5.82 | 282.15 c±11.61 | 190.66 b±17.70 |  | 24.22 a±4.25 | 61.97 b±4.01 | 72.33 c±8.92 |
| 2-Butanol | Nd | Nd | 14.73 a±3.20 |  | Nd | 6.50 a±0.20 | 25.66 b±2.37 |
| 2 Methyl propanol | 6.11 a±0.65 | 54.05 c±5.46 | 32.54 b±4.11 |  | Nd | 3.045 b±0.32 | 0.80 a±0.11 |
| 3 Methyl butanol | 16.35 a±4.88 | 72 c±5.22 | 64 b±8 |  | 7.56a±1.11 | 19.92 b±2.23 | 23.73 c±3.60 |
| Hexanol | 11.06 a±3.51 | 19.41 c±3.65 | 16.37 b±4.43 |  | 16.15 c±2.05 | 10.71 a±2.41 | 15.64 b±3.75 |
| Hexen-3-ol (z) | 7.42 a±0.32 | 13.11 b±3.80 | 17.53 c±4.83 |  | 10.18 a±1.11 | 17.47 c±2.62 | 14.93 b±4.05 |
| Heptanol | Nd | 3.50 a±0.30 | 5.65 b±0.74 |  | Nd | Nd | Nd |
| Benzyl alcohol | Nd | 9.86 a±0.62 | 13.68 b±2.65 |  | Nd | 10.18 a±2.88 | 9.53 a±2.36 |
| Phenylethylalcohol | 10.89 a±2.62 | 43.23 c±2.65 | 38.93 b±5.61 |  | 3.72 a±0.24 | 35.72 b±5.04 | 32.32 b±5.11 |
| *Total amounts* | **88.41±17.80** | **497.31** | **395.10±51.27** |  | **61.83±8.71** | **165.52±19.71** | **194.94±30.27** |
| **Acids** |  |  |  |  |  |  |  |
| Acetic acid | Nd | 8.16 b±2.05 | 14.13 a±3.05 |  | Nd | 4.13±0.24 | 10.05±2.10 |
| Propanoic acid | Nd | Nd | 2.36a±0.53 |  | Nd | Nd | 1.04±0.04 |
| *Total amounts* |  | **8.16±2.05** | **16.49±3.58** |  |  | **4.13±0.24** | **11.09±2.14** |
| **Terpenes** |  |  |  |  |  |  |  |
| 3,7 Dimethyl 1,3,7 octatriene | 3.55 a±0.95 | 10.61 b±2.86 | 17.29 c±4.20 |  | 4.53 a±0.36 | 5.01 a±0.55 | 7.11 b±1.25 |
| Copaene | 6.34 b±1.70 | 17.59 b±2.30 | 6.45 a±5.22 |  | 6.74 b±1.11 | 7.18 b±0.90 | 5.83 a±0.54 |
| Farnesene | 11.06 a±2.64 | 47.32 c±5.54 | 29.86 b±8.42 |  | 28.57 a±4.23 | 36.50 b±6.70 | 41.27 c±7.35 |
| *Total amounts* | **19.95±2.65** | **75.52±10,70** | **52.60±17.84** |  | **39.84±5.70** | **48.68±8.15** | **54.21±9.14** |
| **Volatile phenols** |  |  |  |  |  |  |  |
| Guaiacol | Nd | Nd | Nd |  | Nd | 6.16 a±1.61 | 6.88 a±1.45 |
| Methyl guaiacol | Nd | Nd | 5.26 a±0.44 |  | Nd | 3.87 a±0.47 | 15.101 b±3.65 |
| *Total amounts* |  |  | **5.26±0.44** |  |  | **10.03±2.09** | **21.98±5.10** |
| **Hydrocarbons** |  |  |  |  |  |  |  |
| Octane | 5.98 a±0.61 | 24 c±2.60 | 19.96 b±4.13 |  | 5.60 a±0.36 | 25.05 b±5.74 | 22.61 b±4.04 |
| Styrene | Nd | 16.51 c±3.80 | 6.29 a±1.05 |  | Nd | 2.68 a±0.30 | 2.81 a±0.54 |
| *Total amounts* | **5.98±0,61** | **40.51±6.40** | **26.25±5.18** |  | **5.60±0.36** | **27.73±6.04** | **25.41±4.58** |
