# Supplementary figures and images for: New process for production of fermented black table olives using selected autochthonous microbial resources
Source: Front Microbiol. 2015 Sep 24;6:1007. doi: 10.3389/fmicb.2015.01007 (PMC4585182; doi:10.3389/fmicb.2015.01007)

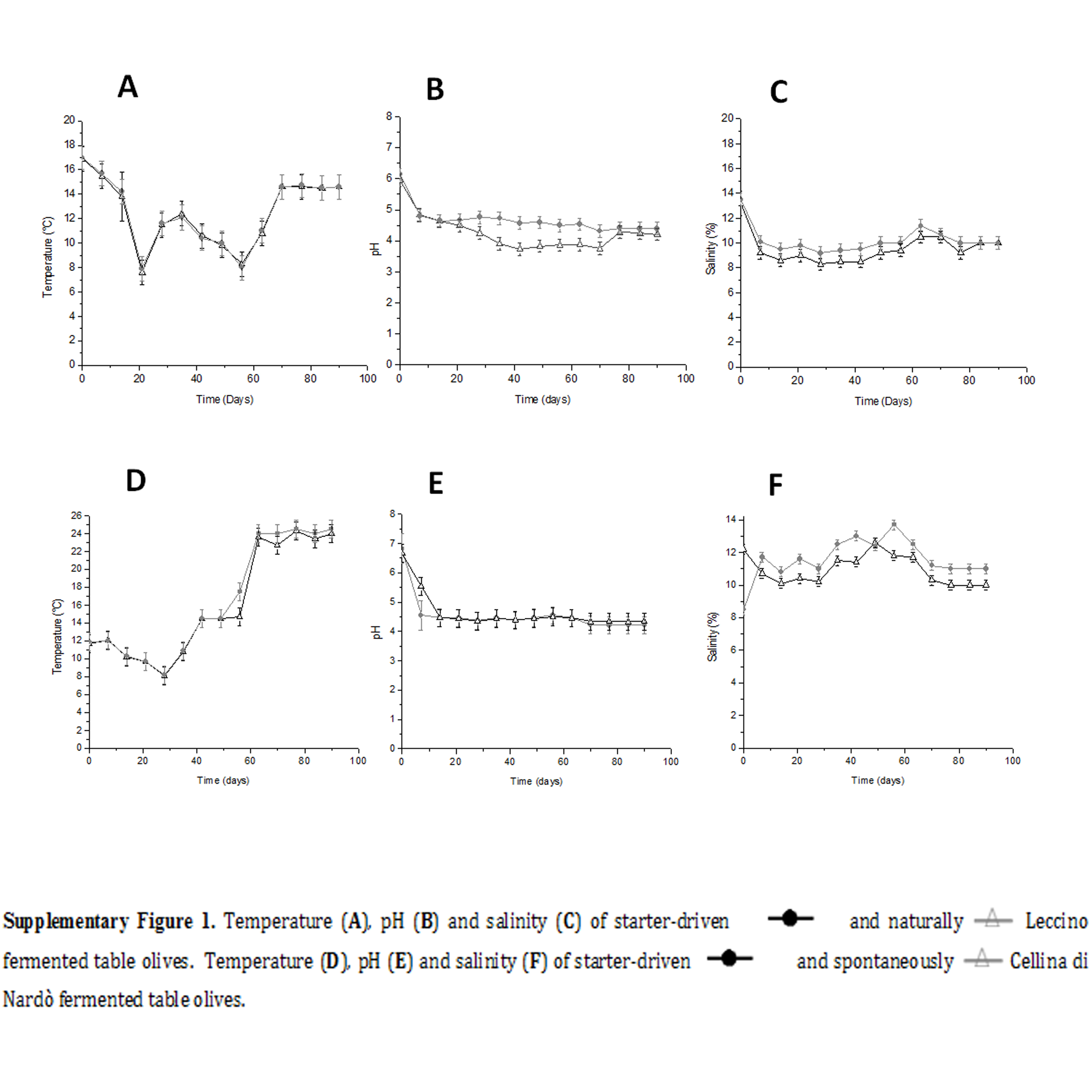

Supplement: Supplementary file 5 [file Image1.JPEG]

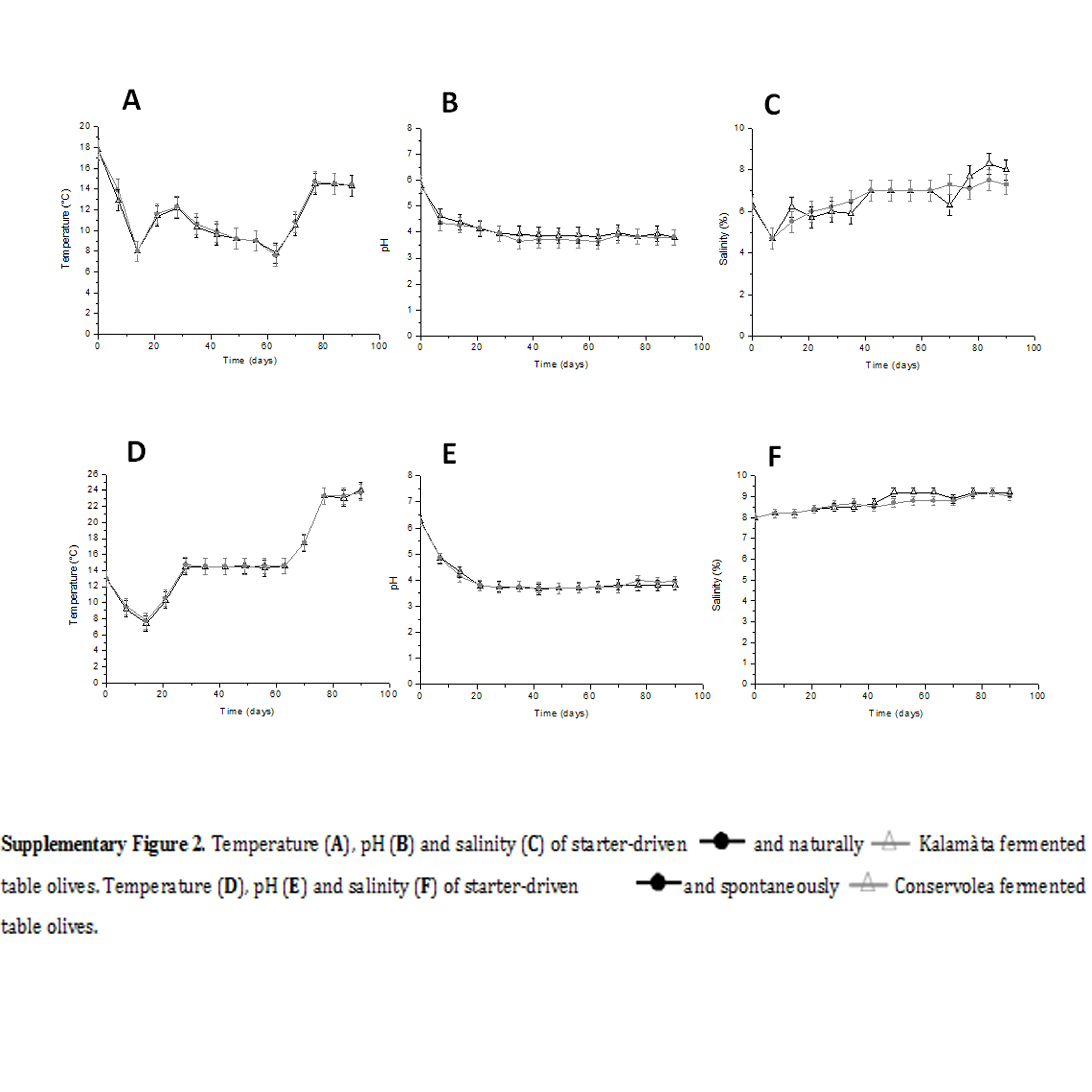

Supplement: Supplementary file 6 [file Image2.JPEG]
